# Supervised Brain Tumor Segmentation Based on Gradient and Context-Sensitive Features

**DOI:** 10.3389/fnins.2019.00144

**Published:** 2019-03-14

**Authors:** Junting Zhao, Zhaopeng Meng, Leyi Wei, Changming Sun, Quan Zou, Ran Su

**Affiliations:** ^1^School of Computer Software, College of Intelligence and Computing, Tianjin University, Tianjin, China; ^2^Tianjin University of Traditional Chinese Medicine, Tianjin, China; ^3^School of Computer Science and Technology, College of Intelligence and Computing, Tianjin University, Tianjin, China; ^4^CSIRO Data61, Sydney, NSW, Australia; ^5^Institute of Fundamental and Frontier Sciences, University of Electronic Science and Technology of China, Chengdu, China

**Keywords:** brain tumor segmentation, gradient, context-sensitive, random forest, mRMR, class-imbalanced

## Abstract

Gliomas have the highest mortality rate and prevalence among the primary brain tumors. In this study, we proposed a supervised brain tumor segmentation method which detects diverse tumoral structures of both high grade gliomas and low grade gliomas in magnetic resonance imaging (MRI) images based on two types of features, the gradient features and the context-sensitive features. Two-dimensional gradient and three-dimensional gradient information was fully utilized to capture the gradient change. Furthermore, we proposed a circular context-sensitive feature which captures context information effectively. These features, totally 62, were compressed and optimized based on an mRMR algorithm, and random forest was used to classify voxels based on the compact feature set. To overcome the class-imbalanced problem of MRI data, our model was trained on a class-balanced region of interest dataset. We evaluated the proposed method based on the 2015 Brain Tumor Segmentation Challenge database, and the experimental results show a competitive performance.

## 1. Introduction

Gliomas, the most common brain tumors in adults, have the highest mortality rate and prevalence among the primary brain tumors (DeAngelis, 2001). They can be classified into high grade gliomas (HGG) and low grade gliomas (LGG). HGG is more aggressive and infiltrative than LGG, thus patients with HGG have a shorter life expectancy (Louis et al., 2007). Magnetic resonance imaging (MRI) with multiple sequences, such as *T*_2_-weighted fluid attenuated inversion recovery (Flair), *T*_1_-weighted (*T*_1_), *T*_1_-weighted contrast-enhanced (*T*_1_c), and *T*_2_-weighted (*T*_2_) provides detailed and valuable information of the brain, and thus is commonly used to diagnose brain diseases, plan the medical treatment strategies, and monitor tumor progression (Bauer et al., 2013; Zeng et al., 2018a). However, gliomas from MRI are difficult to localize as they invade into almost everywhere in the brain with various shapes and sizes and heterogeneous growth patterns (Zhao et al., 2018); they have similar appearances with other diseases such as stroke or inflammation observed in the images; and they are also tangled with surrounding tissues, causing the boundaries diffusive and blurry (Goetz et al., 2016). Furthermore, the scale of MRI voxels is not uniform as the X-ray computed tomography (CT) scans, causing the same tumors to have different gray values, especially when the scans are obtained at different institutions (Sapra et al., 2013). Manual segmentation requires expertise and manually labeling each voxel is laborious and time-consuming (Gordillo et al., 2013). Meanwhile, a variability of 20% and 28% for intra- and inter-rater respectively has been reported for manually segmentation of brain tumors (Mazzara et al., 2004; Goetz et al., 2016). For these reasons, automatic methods instead of manual segmentation with high accuracy and less time-consumption is in high demand.

In this paper, our goal is to propose an automatic method to detect the three different regions of interest (ROI): complete tumor, tumor core, and enhancing tumor from the brain MRI. Our main contributions can be summarized as following:

1. A group of features named circular context-sensitive (CCS) features were proposed. The CCS features fully utilize the histogram information of rays along various orientations and with various lengths.
2. Gradient information was fully utilized by extracting two-dimensional and three-dimensional features. A total of 62 features were extracted to detect and classify the brain tumors.
3. We used an mRMR feature selection algorithm, which could select features that have minimum redundancy and maximum relevance with each others. This is used to significantly reduce the computational cost and increase the efficiency.

The paper is organized as follows: we give a brief literature review of related work in section 2. Then the methods are described in details in section 3. We give the experimental results in section 4, followed by the conclusions in section 5.

## 2. Related Work

Numerous methods of brain tumor detection and segmentation including semi-automatic methods and full-automatic techniques have been proposed (Tang et al., 2017). These segmentation techniques can be roughly divided into 4 categories: threshold-based techniques, region-based techniques, model-based techniques, and pixel/voxel classification techniques.

The threshold-based techniques, region-based techniques, and pixel classification techniques are commonly used for two-dimensional image segmentation (Vijayakumar and Gharpure, 2011). Model-based techniques and voxel classification methods are usually used for three-dimensional image segmentation. We will review the four types of methods in the following subsections.

### 2.1. Threshold-Based Techniques

Threshold-based method is a simple and computationally efficient approach to segment brain tumors because only intensity values need to be considered. The objects in the image are classified by comparing their intensities with one or more intensity threshold values (Gordillo et al., 2013). The Otsu algorithm (Otsu, 1979), Bernsen algorithm (Bernsen, 1986), and Niblack algorithm (Niblack, 1986) are simple and commonly used algorithms.

Gibbs et al. proposed an unsupervised approach using a global threshold to segment. The ROI for the tumor extraction task from the MRI images (Gibbs et al., 1996). Stadlbauer et al. used the Gaussian distribution of intensity values as the threshold to segment tumors in brain *T*_2_-weighted MRI (Stadlbauer et al., 2004). However, if the information in the image is too complex, the threshold-based algorithm is not suitable. It is also limited to extract enhanced tumor areas.

### 2.2. Region-Based Techniques

Region-based methods divide an image into several regions that have homogeneity properties according to a predefined criterion (Adams and Bischof, 1994). Region growing and watershed methods are the most commonly used region-based methods for brain tumor segmentation.

Ho et al. proposed a region competition method which modulates the propagation term with a signed local statistical force to reach a stable state (Ho et al., 2002). Salman et al. examined the seeded region growing and active contour to be compared against experts' manual segmentations (Salman et al., 2012). Sato et al. proposed a Sobel gradient magnitude-based region growing algorithm which solves the partial volume effect problem (Sato et al., 2000). Deng proposed a region growing method which was based on the gradients and variances along and inside of the boundary curve (Deng et al., 2010).

Letteboer et al. and Dam et al. described multi-scale watershed segmentation (Letteboer et al., 2001; Dam et al., 2004). Letteboer et al. proposed a semi-automatic multi-scale watershed algorithm for brain tumor segmentation in MR images (Letteboer et al., 2001). Region-based techniques are used commonly in brain tumor segmentation. However, region-based segmentation has the over-segmentation problem and there is considerable difficulty in marker extraction when using marker-based watershed segmentation. Li and Wan solved these problems by proposing an improved watershed segmentation method with an optimal scale based on ordered dither halftone and mutual information (Li and Wan, 2010).

### 2.3. Model-Based Techniques

Model-based segmentation techniques could be divided into parametric deformable and geometric deformable approaches. There are a number of studies on image segmentation based on active contours, which is a popular parametric deformable method (Boscolo et al., 2002; Amini et al., 2004). Snake is one of the most commonly used geometric deformable algorithm for brain tumor segmentation. Luo et al. proposed a deformable model to segment brain tumors (Luo et al., 2003). This method combined the adaptive balloon force and the gradient vector flow (GVF) force to increase the GVF snake's capture range and convergence speed. Ho et al. proposed a new region competition method for automatic 3D brain tumor segmentation based on level-set snakes which overcome the difficulty in initialization and the missing boundary problems by modulating the propagation term with a signed local statistical force (Ho et al., 2002).

### 2.4. Pixel/Voxel Classification Techniques

Voxel-based classification usually uses voxel attributes for each voxel in the image such as gray level and color information. In brain tumor segmentation, voxel-based techniques are classified as unsupervised classifiers and supervised classifiers to cluster each voxel in the feature space (Gordillo et al., 2013).

Juang and Wu proposed a color-converted segmentation approach with the K-means clustering technique for MRI which converts the input gray-level MRI image into a color space image and the image is labeled by cluster indices (Juang and Wu, 2010). Selvakumar et al. implemented a voxel classification method which combined K-means clustering and fuzzy C-means (FCM) segmentation (Selvakumar et al., 2012). Vasuda and Satheesh improved the conventional FCM by implementing data compression including quantization and aggregation to significantly reduce the dimensionality of the input (Vasuda and Satheesh, 2010). Comparing to the conventional FCM, the modified FCM has a higher convergence rate. Ji et al. proposed a modified possibilistic FCM clustering of MRI utilizing local contextual information to impose local spatial continuity to reduce noise and resolve classification ambiguity (Ji et al., 2011). Autoencoders were used in Vaidhya et al. and Zeng et al. work for brain tumor segmentation and other imaging tasks (Vaidhya et al., 2015; Zeng et al., 2018b). Zhang et al. proposed a hidden Markov random field model and the expectation-maximization algorithm for brain segmentation on MRI (Zhang et al., 2001).

For the voxel-classification MRI processing techniques, proper depiction of voxels is required as a criteria to accurately classify each voxel. In the previous studies, Zulpe et al. used gray-level co-occurrence matrix (GLCM) textural features to detect the brain tumors (Zulpe and Pawar, 2012); Context-sensitive features were used in Meier et al.'s study to classify tumors and non-tumors (Meier et al., 2014). Meanwhile, a feature selection algorithm also requires good designs to select a compact set of features in order to reduce the computation cost (Zou et al., 2016a,b; Su et al., 2018), considering the huge data size of the MRI. In our study, one set of informative features and efficient feature selection algorithm were proposed. The experimental results have demonstrated that promising brain tumor segmentation performance can be achieved using the proposed method.

## 3. Methodology

**Figure 1:**
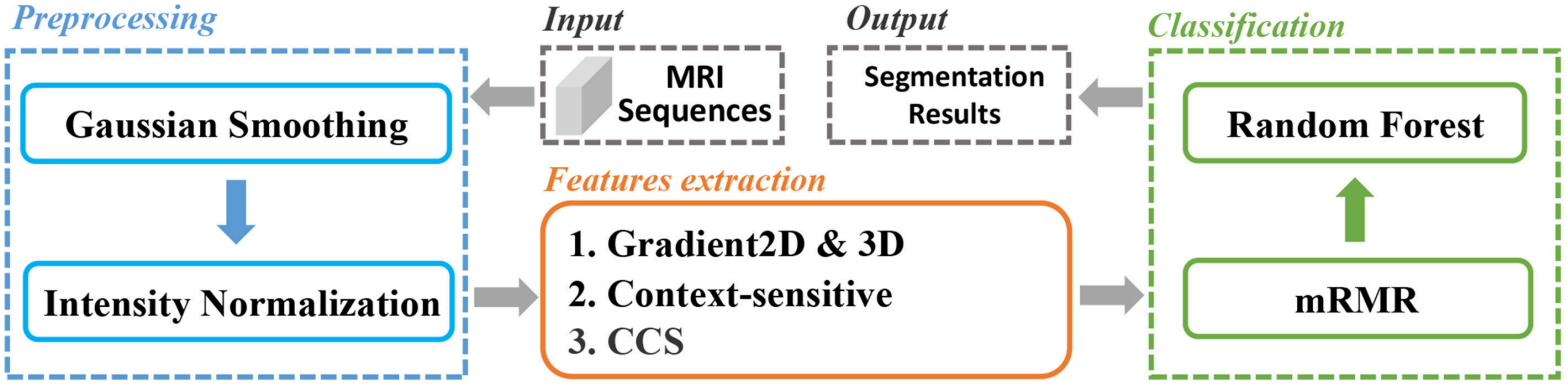
The pipeline of our proposed method.

In this paper, we extracted various types of features from the brain MRI and used for classification. And an mRMR feature selection method was used to reduce the feature dimension and select the best feature set. The whole pipeline was depicted in Figure 1. Firstly, the MRI sequences were pre-processed with smoothing and normalization operations. Secondly, we extracted two types of features, gradient-based features and context-sensitive features. Thirdly, we used an mRMR feature selection method to select the optimal feature set with minimal redundancy and maximal relevance. We will explain the whole process in detail later.

### 3.1. Data

We used the training data of BraTS 2015 as our training and test data (Menze et al., 2015). It provides 4 sequences *T*_1_, *T*_1_*c*, *T*_2_, and Flair. The image data contains 220 HGG (anaplastic astrocytomas and glioblastoma multiforme tumors) MR scans and 54 LGG (histological diagnosis: astrocytomas or oligoastrocytomas) cases. The “ground truth” are labeled by manual annotations with 0-5 with four types of tumoral structures labeled as the following: “necrotic (or fluid-filled) core” is labeled 1, “edema” is labeled 2, “non-enhancing(solid) core” is labeled 3, and “enhancing core” is labeled 4. The normal tissue is labeled 0. We evaluated our work within three regions: complete tumor (which contains necrotic core, edema, non-enhancing core and enhancing core), tumor core (which contains necrotic core, non-enhancing, and enhancing core) and enhancing tumor.

### 3.2. Pre-processing

We carried out smoothing and normalization on the MRI sequences to reduce the impact of image noise and to enhance image quality for further processing. As for smoothing, we chose the Gaussian filter which has been widely used in image processing and computer vision for noise suppression (Bergholm, 1987; Deng and Cahill, 1993; Kharrat et al., 2009; Zeng et al., 2017).

For further processing, MRI sequences are sensitive to all the acquisition conditions such as MR protocols, MR scanners, and MR adjustments (Sled et al., 1998). Even for the same tissue information acquired with the same conditions, there will be a variation because MRI intensities do not have a tissue specific value. In order to eliminate the impact of the variation for further image processing which is based on image intensity, we normalized the smoothed value to the range from 0 to 1. The normalization was calculated as in Equation (1)

(1) $$\begin{array}{r} X^{*}=\frac{X-X_{\text{min}}}{X_{\text{max}}-X_{\text{min}}} \end{array}$$

where X^*^ and *X* are the normalized and raw gray value respectively; *X*_max_ is the maximal gray value, and *X*_min_ is the minimal gray values.

### 3.3. Gradient Based Features

The gradient value represents the rate of change in the direction of the largest possible intensity change. In our study, we used the central difference gradient as the gradient operator. For each voxel *p*, the derivative at one direction is the mean of the two voxels adjacent to *p* in that particular direction. Here we calculated two sets of gradient-based features within the ROI. The first set calculated the gradient along each coordinate plane, which we named as Gradient2D. The Gradient2D of one image in each coordinate plane has two components: the x-derivative and the y-derivative. In Equation (2), we take the x-derivative as an example (*I* is the input image). The second set, the Gradient3D, is based on the three-dimensional gradient magnitude. We further divide the Gradient3D into five subsets, the GM, rMean, rVar, seqMean, and seqVar, and we show them in Table 1.

**Table 1:** The five feature subsets in Gradient3D.

**Feature name**	**Modality**	**Cube size**
GM	Flair, *T*_1_, *T*_1_*c*, *T*_2_	–
rMean	Flair, *T*_1_, *T*_1_*c*, *T*_2_	3, 5, 7
rVar	Flair, *T*_1_, *T*_1_*c*, *T*_2_	3, 5, 7
seqMean	all modalities	3, 5, 7
seqVar	all modalities	3, 5, 7

The GM feature consists of the three-dimensional gradient magnitude, which is calculated based on Equation (3). *G*_*x*_ is the directional gradient along the x-axis, *G*_*y*_ is the directional gradient along the y-axis and *G*_*z*_ is the directional gradient along the z-axis. In our study, we also used the central difference gradient as gradient operator to extract the GM feature for each respective MRI image sequence. The operator is given in Equation (2).

(2) $$\begin{array}{r} dI/dx=\frac{I\left(x+1\right)-I\left(x-1\right)}{2} \end{array}$$

(3) $$\begin{array}{r} mag\left(G_{x},G_{y},G_{z}\right)=\sqrt{G_{x}^{2}+G_{y}^{2}+G_{z}^{2}} \end{array}$$

We further extracted the rMean and rVar features by calculating the mean and variance of the GM feature over a cube-shaped neighborhood with sizes 3^3^, 5^3^, 7^3^ for each GM feature of each respective sequence. Meanwhile, we extracted seqMean and seqVar by calculating the mean and variance of the GM features over the sequences in cube-shaped neighborhoods.

### 3.4. Circular Context-Sensitive Feature

Meier et al. proposed context-sensitive features for brain tumor segmentation which extracts ray features in plane by calculating the histogram using intensity values from *T*_1_ and Flair-weighted images after atlas-normalization (Meier et al., 2014). The rationale of this method is that the intensity range of *T*_1_ and Flair-weighted modalities is larger than that of the healthy tissues. Based on this method, we proposed a circular context-sensitive (CCS) feature to capture more details in various sizes.

**Figure 2:**
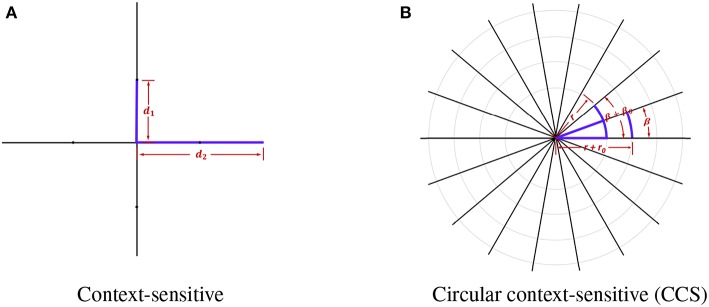
The original context-sensitive features and the circular context-sensitive (CCS) features. **(A)** shows the original context-sensitive features. **(B)** shows the CCS features. In this example, the center voxel sends out 18 rays of length *r*+n^*^r__θ__ with an angle β+n^*^β_θ_. The CCS can fully extract context information instead of only voxel information in horizontal or vertical directions.

In context-sensitive features, every voxel sends out four rays with radius *r* ∈ {10, 20} and orienting at *ang* ∈ {0, π/2, π, 2π/3}. In order to obtain more information and extract features in multiple scales, we made several improvements to the original context-sensitive features. Firstly, instead of utilizing only voxel information in the horizontal or vertical directions, we used rays evenly distributed on a circle to swipe all the orientations, which are denser. The directions are calculated using the following equation:

(4) $$\begin{array}{l} \begin{array}{c} ang=\beta +n*\beta_{\theta },n\in N,\\ \;\beta_{\theta }=2\pi /N_{\beta } \end{array} \end{array}$$

where β is the initial angle, β_θ_ is the step size rotating around the center point, and *N*_β_ is the total number of directions. Secondly, in order to capture context features with all the scales, we used a continuous radius to cover the neighboring voxel information as much as possible. The radius *r* is defined as:

(5) $$\begin{array}{l} \begin{array}{c} r=r_{0}+n*r_{{}_{\theta }},n\in N^{*},\\ r_{\theta }=\left(r_{\text{max}}-r_{\text{min}}\right)/N_{r} \end{array} \end{array}$$

where *r*_0_ is the initial radius, where *r*_θ_ is the step size moving toward the outermost circle, *r*_min_ and *r*_max_ are the minimum and maximum of the radius, and *N*_*r*_ is the total number of rays. We show the comparison between the original context-sensitive features and the circular context-sensitive features in Figure 2. The original context-sensitive sends out four rays in four directions. The CCS features, however, send out rays along all the orientations and with all the radius, which is supposed to capture rich context information. In our studies, we used 8 rays evenly distributed on 45 circles ranging from 10 to 20 with even numbers to capture the context-sensitive features.

In the MRI, the slice thickness varies considerably which will affect the feature extraction results so the features are considered only for the *T*_1_ and Flair-weighted in-plane images. In summary, our CCS feature extraction is summarized as the following:

1. For one voxel *i*, we calculated *r* and *ang* of all the rays, based on Equations (4, 5).
2. Using *T*_1_ and Flair images, we computed the histograms and obtained the maximal *H*_max_ and minimal *H*_min_ histogram values.
3. The mean values of each ray was calculated using *H*_max_ and *H*_min_ as the CCS feature values.

In summary, we extracted 12 Gradient2D features, 34 Gradient3D features, 4 context-sensitive features, and 12 CCS features, as shown in Table 2. In the next section, we will show the voxel-based classification and feature selection to label different regions of the brain MR images.

**Table 2:** Number of features in each feature group.

**Feature type**	**Counts**
Gradient2D	12
Gradient3D	34
Context-sensitive	4
CCS	12
Total	62

### 3.5. Feature Selection Based on mRMR

We classified the brain MRI images into five categories: normal tissue, edema, non-enhancing core, necrotic core, and enhancing core. Random forests (RF) (Breiman, 2001) is an ensemble learning method for classification, regression, and other tasks, and has been widely used in image analysis (Jin et al., 2014; Liu et al., 2015). It consists of multitude of decision trees and outputs the votes over each tree. They are able to handle multi-class problems, and they provide a probabilistic output instead of hard label separations. However, due to the large volume of MRI images, direct classification based on the extracted features (as shown in Table 2) is time-consuming. A proper feature selection algorithm will greatly reduce the computational cost and increase the efficiency.

Minimal Redundancy maximal Relevance(mRMR) was proposed by Peng et al. which can select features that have minimum redundancy and maximum relevance with each other (Peng et al., 2005). We use Equation (6) to search for features which have the maximum relevance, and use the minimal redundancy condition as the Equation (7) to select mutually exclusive features. Equation (8) gives mRMR features. *x*_*i*_ represents the *i*−th feature in feature set *S* with target class *c*. Φ means the combination of *D* and *R*.

(6) $$\begin{array}{r} maxD\left(S,c\right),D=\frac{1}{\left|S\right|}\underset{x_{i}\epsilon S}{\sum }I\left(x_{i};c\right) \end{array}$$

(7) $$\begin{array}{r} minR\left(S\right),R=\frac{1}{|S{|}^{2}}\underset{x_{i},x_{j}\epsilon S}{\sum }I\left(x_{i},x_{j}\right) \end{array}$$

(8) $$\begin{array}{r} max\Phi \left(D,R\right),\Phi =D-R \end{array}$$

In order to avoid over-learning and under-learning, we evaluated our RF classifier by 5-fold cross validation with the measurements in section 3.7. In detail, we divided the subcases into 5 roughly equal parts. For each *k* = 1, 2, …, 5, we fit the RF model to the other 4 parts, and predict the *k*th part with the fitted RF model. The final outcome equals to the mean of the results of the 5-folds. In our study, we used the mRMR feature selection method to select the minimal feature set which reduces the computational cost without performance degradation. The raw 62-dimensional feature set is shown in Table 2. The mRMR feature selection details are as follows:

1. Train the RF classifier with top *f* features which are ranked by mRMR from the raw feature set and test the model with 5-fold cross validation. Here, we set *f* = 62−5*n, n* = 0, 1, 2, …, 12.
2. Rank the performance in step 1. Set the final feature set with *f*_*m*_ dimension as the features which achieve the best performance among all the *f*-dimensional features. Here, *f*_*m*_ is the dimension of the final feature set.

### 3.6. Solution to the Class-Imbalance Problem

The “BraTS” data is seriously unbalanced, with less than 1% of voxels being tumor voxels. Training on them would result in problems such as higher mis-classification rate for the minority class data. Thus, we carried out three steps to overcome the class-imbalance problem.

1. Detect the boundary of ROI: We used a plane to move along each axis's direction until it detected a voxel labeled with none-zero. Then the plane in the current position was used as the boundary in that direction.
2. Split the ROI: After step 1, each raw MR image would have a total of 6 boundaries in the xyz space. We split out a cuboid as our ROI using the boundaries. This removed a large number of zero label voxels and voxels inside each ROI contained all the categories.
3. Equally select voxels for each category: Within the segmented ROI, the label with the least number of voxels was recorded. We randomly picked the equal number of voxels in the ROI to form a balanced data.

### 3.7. Performance Measurements

To show the performance of our segmentation approach, we use Dice, positive predictive value (PPV), Sensitivity, Specificity to evaluate HGG and LGG tumor regions segmentation. TP represents the number of “true positive,” where “true positive” is the event that the test makes a positive prediction, and the subject has a positive ground truth. FP is the number of “false positive,” where “false positive” is the event that the test makes a positive prediction, and the subject has a negative ground truth. FN is the number of “false negative” and TN indicateds the size of “true negative” set.

1. Dice
  $$Dice\;=\frac{TP}{\left(\left(TP+FP\right)+\left(TP+FN\right)\right)/2}$$
  The Dice score normalizes the number of true positives to the average size of the two segmented areas. It is identical to the F-score (the harmonic mean of the precision recall curve) and can be transformed monotonously to the Jaccard score.
2. Positive predictive value (PPV)
  $$PPV\;=\frac{TP}{TP+FP}$$
  The PPV represents the proportions of positive results in tests that are true positive results.
3. Sensitivity
  $$Sensitivity\;=\frac{TP}{TP+FN}$$
  Sensitivity measures the proportion of actual positives that are correctly identified as such.
4. Specificity
  $$Specificity\;=\frac{TN}{FP+TN}$$
  Specificity (also called the true negative rate) measures the proportion of actual negatives that are correctly identified as such.

## 4. Experimental Results

In our experiments, we extracted four groups of features after pre-processing: Gradient2D features, Gradient3D features, context-sensitive features, and CCS features, including totally 62 features (as shown in Table 2). The Gradient3D set contains five subsets: the GM, rMean, rVar, seqMean, and seqVar. We used the mRMR feature selection method to select a compact set of features and built the random forest classifier.

Firstly, we built the random forest classifier using the top *f* feature set ranked by mRMR respectively. We used *f* to denote the dimension of the feature set, where *f* = 62−5*n, n* = 0, 1, 2, …, 12. Secondly, we compared the performance of feature sets with diverse *f*. The best feature dimension *f*_*m*_ is the *f* which has the best performance. We recorded this *f*_*m*_-dimensional feature set as the optimal feature set. Here we present the performance comparisons between diverse *f*s; Then we show the performance comparisons among different feature groups. Next, we compared our results with Meier et al.'s method (Meier et al., 2014). Lastly, we show the segmentation results marked with different colors.

### 4.1. Comparison Between Feature Sets With Different Dimensions

In this step, we trained our model with different numbers of features as shown in Table 3. For the *n*-th training, we selected the top *f*-dimensional feature set ranked by the mRMR feature selection method according to their relevance and redundancy, where *f* = 62−5*n, n* = 0, 1, 2, …, 12. Here we used random forest as our classifier and set the number of trees in the forest to 100. For every *n*, we evaluated the model by the measurements in section 3.7 within three regions: complete tumor, tumor core, and enhancing tumor. The HGG&LGG, HGG, and LGG MR scans subsets were tested.

**Table 3:** Performance of feature sets with different dimension.

**The nth training**	**Feature Dimension**	**Measurements**	**Dice**			**PPV**			**Sensitivity**			**Specificity**		
		**ROI**	**Complete**	**Core**	**Enhancing**	**Complete**	**Core**	**Enhancing**	**Complete**	**Core**	**Enhancing**	**Complete**	**Core**	**Enhancing**
1	62	HGG&LGG	0.91	0.62	0.61	0.94	0.63	0.64	0.89	0.61	0.58	0.79	0.81	0.92
		HGG	0.92	0.63	0.61	0.94	0.65	0.64	0.89	0.62	0.59	0.79	0.82	0.92
		LGG	0.86	0.54	–	0.91	0.56	–	0.83	0.55	–	0.76	0.55	–
2	57	HGG&LGG	0.91	0.62	0.61	0.94	0.63	0.64	0.88	0.61	0.58	0.78	0.80	0.92
		HGG	0.92	0.63	0.61	0.94	0.64	0.64	0.89	0.61	0.58	0.79	0.82	0.92
		LGG	0.87	0.55	–	0.91	0.55	–	0.84	0.57	–	0.75	0.57	–
3	52	HGG&LGG	0.91	0.62	0.61	0.94	0.63	0.64	0.88	0.61	0.58	0.78	0.80	0.92
		HGG	0.91	0.63	0.61	0.94	0.64	0.64	0.89	0.61	0.58	0.79	0.82	0.92
		LGG	0.87	0.55	–	0.91	0.55	–	0.83	0.57	–	0.74	0.57	–
4	47	HGG&LGG	0.91	0.62	0.61	0.94	0.63	0.64	0.88	0.61	0.58	0.78	0.80	0.92
		HGG	0.91	0.63	0.61	0.94	0.64	0.64	0.89	0.61	0.58	0.79	0.81	0.92
		LGG	0.87	0.56	–	0.91	0.55	–	0.83	0.57	–	0.74	0.57	–
5	42	HGG&LGG	0.91	0.62	0.61	0.94	0.63	0.64	0.88	0.61	0.58	0.78	0.80	0.92
		HGG	0.91	0.63	0.61	0.94	0.64	0.64	0.89	0.61	0.58	0.79	0.81	0.92
		LGG	0.87	0.55	–	0.91	0.55	–	0.83	0.56	–	0.74	0.56	–
6	37	HGG&LGG	0.91	0.61	0.60	0.94	0.63	0.64	0.88	0.61	0.58	0.78	0.80	0.92
		HGG	0.91	0.63	0.60	0.94	0.64	0.64	0.89	0.61	0.58	0.79	0.82	0.92
		LGG	0.86	0.55	–	0.92	0.56	–	0.82	0.54	–	0.76	0.54	–
7	32	HGG&LGG	0.91	0.62	0.61	0.94	0.62	0.64	0.88	0.61	0.58	0.76	0.79	0.92
		HGG	0.91	0.63	0.61	0.94	0.64	0.63	0.89	0.62	0.58	0.77	0.80	0.92
		LGG	0.87	0.55	–	0.91	0.55	–	0.83	0.57	–	0.72	0.57	–
8	27	HGG&LGG	0.91	0.62	0.61	0.93	0.62	0.64	0.89	0.62	0.58	0.76	0.78	0.92
		HGG	0.91	0.63	0.61	0.94	0.64	0.63	0.89	0.62	0.58	0.77	0.80	0.92
		LGG	0.87	0.55	–	0.90	0.54	–	0.83	0.57	–	0.71	0.57	–
9	22	HGG&LGG	0.91	0.62	0.61	0.93	0.62	0.64	0.89	0.62	0.58	0.75	0.77	0.92
		HGG	0.91	0.63	0.61	0.94	0.63	0.63	0.89	0.63	0.58	0.76	0.79	0.92
		LGG	0.87	0.56	–	0.90	0.54	–	0.84	0.58	–	0.71	0.58	–
10	17	HGG&LGG	0.91	0.62	0.61	0.93	0.61	0.64	0.89	0.62	0.58	0.73	0.76	0.92
		HGG	0.91	0.63	0.61	0.93	0.62	0.64	0.89	0.63	0.58	0.74	0.78	0.92
		LGG	0.86	0.55	–	0.90	0.54	–	0.84	0.57	–	0.70	0.57	–
11	12	HGG&LGG	0.91	0.61	0.60	0.92	0.59	0.63	0.89	0.63	0.57	0.70	0.74	0.92
		HGG	0.91	0.62	0.60	0.93	0.61	0.63	0.90	0.63	0.57	0.71	0.76	0.91
		LGG	0.87	0.56	–	0.89	0.54	–	0.85	0.59	–	0.67	0.59	–
12	7	HGG&LGG	0.90	0.58	0.55	0.91	0.56	0.59	0.89	0.61	0.52	0.67	0.70	0.91
		HGG	0.90	0.59	0.55	0.92	0.57	0.59	0.89	0.62	0.52	0.68	0.72	0.91
		LGG	0.86	0.54	–	0.88	0.52	–	0.84	0.56	–	0.65	0.56	–
13	2	HGG&LGG	0.86	0.48	0.34	0.85	0.44	0.36	0.87	0.53	0.33	0.42	0.52	0.86
		HGG	0.87	0.48	0.36	0.86	0.44	0.38	0.89	0.54	0.34	0.42	0.54	0.86
		LGG	0.81	0.43	–	0.83	0.44	–	0.80	0.42	–	0.41	0.42	–

**Figure 3:**
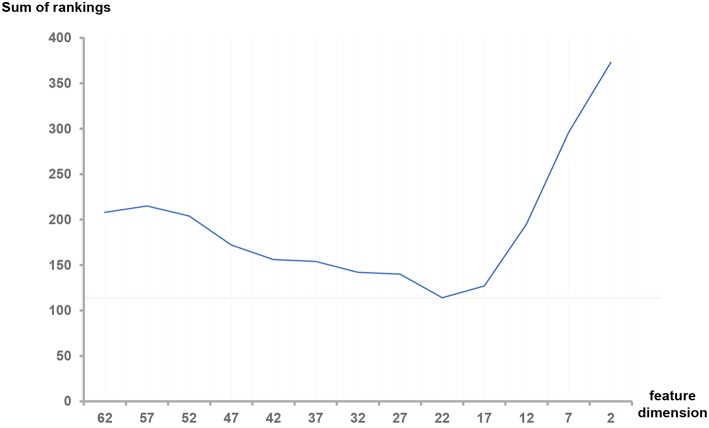
Sum of rankings for each feature dimension *f*. The ranking rules are defined as follows: In each feature set with dimension *f*, we observed the three metrics (Dice, sensitivity and specificity) in three ROIs (Complete, enhancing and core) of HGG, LGG and both, totally 32 values in Table 3. We ranked the corresponding values across all the *f*. If equal values appeared, we took another metric, the size of the feature set into account. Then we summed all the rankings of each *f* and plotted the curve.

As shown in Table 3, the difference between the results of two adjacent experiments is very small. It is difficult to distinguish which dimension *f* has the best performance. In order to obtain an intuitive feature selection outcome, we provide an overall ranking of the performances for each *f* and show the results in Figure 3. It can be seen that 22-dimensional feature set achieves the best performance among all the tested feature sets.

### 4.2. Comparison Between Different Feature Groups

In our studies, We have tested four different types of features. In order to learn which feature group is more informative for classification, we trained the model with each feature group and compared the performances of each feature group in Table 4. The table shows that the Gradient3D group performed far better than the other groups. It obtained a high Dice score (0.91) for complete tumor in HGG and a high PPV (0.92) for complete tumor in HGG&LGG datasets. And the CCS features performs slightly better than the context-sensitive features. However, compared with using all the 62 features, using a single group cannot achieve a better performance, which shows that each group is useful for classification and the integration of all the four groups is more helpful for classification.

**Table 4:** Performance of different feature groups.

**Feature type**	**Feature Dimension**	**Measurements**	**Dice**			**PPV**			**Sensitivity**			**Specificity**		
		**ROI**	**Complete**	**Core**	**Enhancing**	**Complete**	**Core**	**Enhancing**	**Complete**	**Core**	**Enhancing**	**Complete**	**Core**	**Enhancing**
Gradient2D	12	HGG&LGG	0.89	0.54	0.41	0.87	0.47	0.49	0.90	0.63	0.35	0.50	0.56	0.92
		HGG	0.89	0.55	0.40	0.88	0.48	0.49	0.90	0.64	0.34	0.51	0.57	0.91
		LGG	0.85	0.54	–	0.84	0.48	–	0.87	0.62	–	0.47	0.62	–
Gradient3D	34	HGG&LGG	0.90	0.61	0.60	0.92	0.60	0.63	0.89	0.62	0.57	0.71	0.75	0.92
		HGG	0.91	0.62	0.60	0.93	0.61	0.63	0.90	0.63	0.57	0.72	0.76	0.91
		LGG	0.85	0.51	–	0.88	0.50	–	0.83	0.55	–	0.62	0.55	–
Context-sensitive	4	HGG&LGG	0.86	0.51	0.14	0.87	0.44	0.23	0.85	0.60	0.11	0.51	0.48	0.91
		HGG	0.87	0.51	0.16	0.87	0.44	0.24	0.86	0.60	0.12	0.51	0.47	0.90
		LGG	0.79	0.45	–	0.83	0.43	–	0.76	0.47	–	0.52	0.47	–
CCS	12	HGG&LGG	0.86	0.54	0.13	0.89	0.47	0.25	0.84	0.62	0.09	0.62	0.57	0.94
		HGG	0.87	0.53	0.16	0.90	0.48	0.27	0.85	0.61	0.11	0.62	0.56	0.92
		LGG	0.80	0.48	–	0.86	0.47	–	0.75	0.48	–	0.64	0.48	–

### 4.3. Comparison With Other Method

We compared our methods with another method which was proposed by Meier et al. (2014). They extracted appearance-sensitive and context-sensitive features and also used random forest as a classifier.

As shown in Table 5, Meier et al. evaluated the classifier with three ROIs: complete tumor, tumor core, and enhancing tumor. However, the LGG performance was not mentioned. In our experiments, we trained the HGG&LGG, HGG, and LGG models and tested our models with three ROIs. As shown in Table 3, we had better performance especially for complete tumor. Compared to Meier et al.'s method, we not only extract the context-sensitive features, but we also made improvements and proposed the circular context-sensitive features, which considered multiple scales and multiple directions.

**Table 5:** Comparison between Meier et al.'s method and the proposed method.

**Method**	**Measurements**	**Dice**			**PPV**			**Sensitivity**			**Specificity**		
	**ROI**	**Complete**	**Core**	**Enhancing**	**Complete**	**Core**	**Enhancing**	**Complete**	**Core**	**Enhancing**	**Complete**	**Core**	**Enhancing**
Meier et al.;s method	HGG&LGG	0.83	0.66	0.58	0.85	0.74	0.66	0.83	0.66	0.54	–	–	–
	HGG	0.84	0.73	0.68	0.80	0.80	0.72	0.89	0.70	0.70	–	–	–
The proposed method	HGG&LGG	0.91	0.62	0.61	0.93	0.62	0.64	0.89	0.62	0.58	0.75	0.77	0.92
	HGG	0.91	0.62	0.61	0.93	0.62	0.64	0.89	0.62	0.58	0.75	0.77	0.92
	LGG	0.87	0.56	–	0.90	0.54	–	0.84	0.58	–	0.71	0.58	–

### 4.4. Performance of Brain Tumor Segmentation

**Figure 4:**
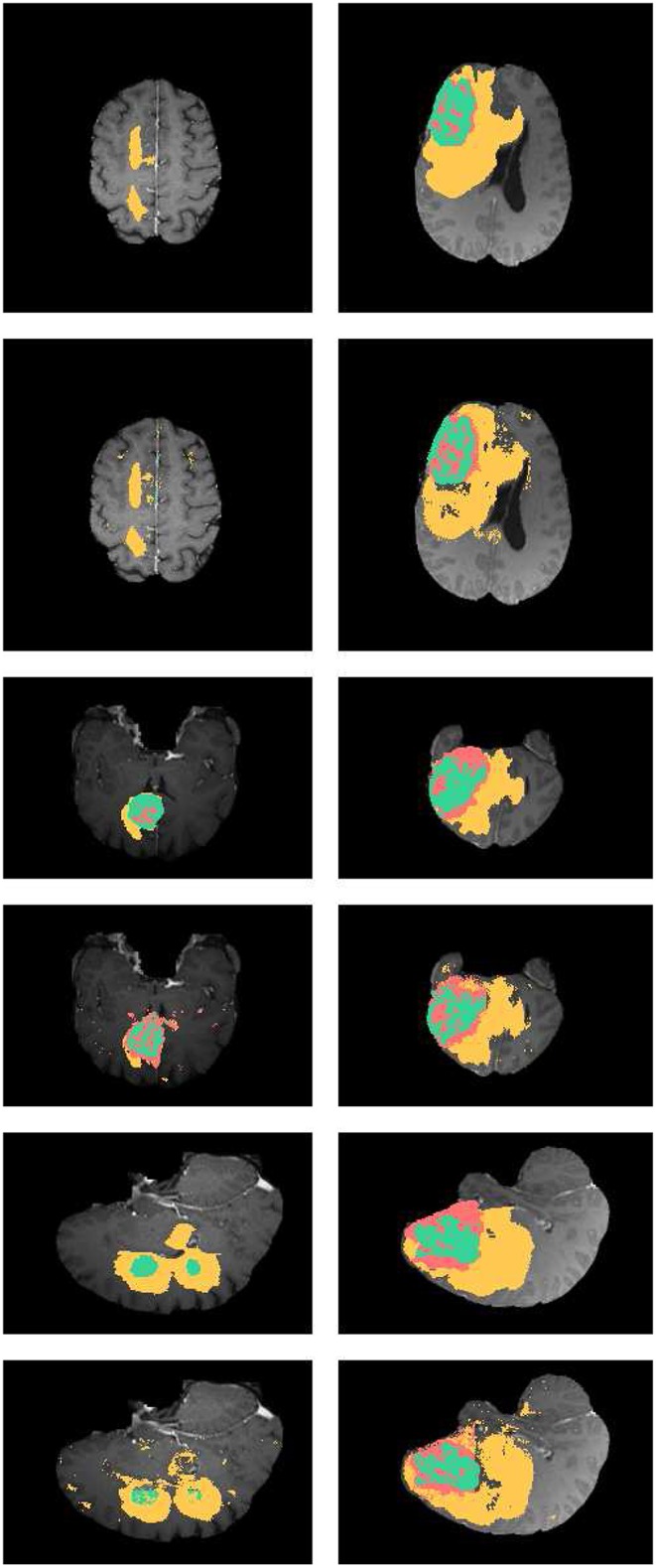
Examples the brain tumor segmentation results using the proposed method. The rows 1,3,5 are the axial, sagittal, and coronal slices of the ground truth. Rows 2,4,6 are the axial, sagittal, and coronal slices of our results. The labels of the tumor structure: enhancing tumor (green), tumor core(green and red), complete tumor (green, red, and yellow).

In Figure 4, the axial, sagittal, and coronal slices of the ground truth are shown in rows 1, 3, and 5, respectively. The corresponding slices of the segmentation results are shown in rows 2, 4, and 6, respectively. As shown in Figure 4, our segmentation results are consistent with the ground truth. And we have good performance in all axial, sagittal, and coronal directions.

## 5. Conclusions

In our study, we proposed a supervised brain tumor segmentation method for MRI scans. We extracted four types of feature groups named Gradient2D set, Gradient3D set, context-sensitive features, and circular context-sensitive features, totally 62 features. Then we selected a set of the most informative feature set based on the mRMR algorithm and used them to build the random forest in order to distinguish different regions of brain tumors. We presented the performance comparisons among different dimensions of feature sets for feature selection, comparisons among different feature subgroups and comparisons with other tumor segmentation approaches. The results show that the proposed method is competitive in segmenting brain tumors.

## Data Availability

Publicly available datasets were analyzed in this study. This data can be found here: http://braintumorsegmentation.org/.

## Author Contributions

JZ conducted the experiements. ZM, LW, and CS participated the manuscript writing. QZ and RS designed the experiments and edited the manuscript.

### Conflict of Interest Statement

The authors declare that the research was conducted in the absence of any commercial or financial relationships that could be construed as a potential conflict of interest.
